# Lipid metabolic adaptations during inflammation are controlled by the circadian clock and impaired by light at night

**DOI:** 10.1007/s00011-025-02066-9

**Published:** 2025-06-30

**Authors:** Beata Benedikova, Viera Sebenova Jerigova, Michal Zeman, Monika Okuliarova

**Affiliations:** Department of Animal Physiology and Ethology, Faculty of Natural Sciences, Comenius University, Bratislava, Slovakia

**Keywords:** Adipose tissue, Circadian rhythms, Inflammation, Light at night, Liver, Metabolism

## Abstract

**Objective and design:**

Immune defence requires systemic metabolic changes to redirect energy and nutrients to activated immune cells. The circadian clock is known to control the immune response, but its role in regulating metabolic adaptations following the immune challenge remains poorly understood. We aimed to examine the inflammatory and metabolic responses in rat liver and visceral white adipose tissue (vWAT) after time-of-day-dependent endotoxin stimulation under a regular light/dark cycle or dim artificial light at night (ALAN; ~2 lx), which disrupts immune and metabolic rhythms. Male rats were challenged with lipopolysaccharide (LPS) either during the day or night and acute changes in metabolic pathways and the peripheral metabolic clocks were analysed at both systemic and molecular levels.

**Results:**

In the control light/dark cycle, we observed higher fatty acid (FA) mobilization in vWAT after daytime than nighttime LPS injection. Similarly, hepatic glucose metabolism was more responsive to daytime than nighttime LPS, while the opposite trend was observed for FA uptake and synthesis. This daily variability in metabolic changes was associated with the inflammatory response, involving nuclear factor interleukin-3 regulated (NFIL3) in the liver and nuclear factor-kappa B (NF-κB)/NLR family, pyrin domain containing 3 (NLRP3) inflammasome pathway in vWAT. Hepatic and adipose clocks also showed time-of-day-dependent response to LPS, indicating a direct link to circadian regulation. Disruption of metabolic clocks by ALAN impaired the capacity of rats to maintain lipid metabolic adaptations during inflammation.

**Conclusions:**

Together, our results highlight the role of circadian clocks in LPS-induced responses of glucose and FA metabolism and their susceptibility to disruption by ALAN.

**Supplementary Information:**

The online version contains supplementary material available at 10.1007/s00011-025-02066-9.

## Introduction

Immune defence is a highly energy-demanding process that requires systemic metabolic changes to redirect energy substrates to activated immune cells, which also adapt their metabolic programs [1, 2]. These metabolic changes are dynamic over time and vary depending on the type of immune attack. Generally, the acute inflammatory response to endotoxin is associated with an increase in plasma glucose and lipid metabolites, primarily regulated by crosstalk between the liver and adipose tissue [3]. Enhanced lipolysis in adipose tissue raises levels of free fatty acids (FAs) in the circulation, from which they are taken up by other organs, including the liver [4, 5]. Additionally, re-esterification of free FAs in the liver significantly contributes to hypertriglyceridemia during inflammation [5, 6]. At the molecular level, substantial transcriptional control of the inflammation-modulated metabolic genes is attributed to lipid-sensing nuclear receptors and transcription factors, particularly peroxisome proliferator-activated receptors alpha (PPARα) and gamma (PPARγ), liver X receptor α, and sterol regulatory element binding protein-1c (SREBP-1c), all of which are downregulated during the acute phase response [7]. For instance, reduced PPARα levels are associated with diminished FA oxidation, while reduced adipose PPARγ levels are related to decreased fat storage [6].

Accumulating evidence indicates that immune defence mechanisms exhibit differential responsiveness based on the timing of antigen exposure [8]. In rats, such daily variability in the inflammatory response has been demonstrated after daytime/nighttime lipopolysaccharide (LPS) injection for the circulating white blood cell (WBC) counts [9], plasma cytokine levels, body temperature [10], and locomotor activity [11]. Daily rhythms of most physiological and behavioural processes, including numerous immune variables, are governed by circadian clocks that maintain their temporal synchrony with external environmental cycles and mutual internal synchrony across the body [12]. The central oscillator in the suprachiasmatic nuclei of the hypothalamus and countless peripheral oscillators share a common molecular mechanism comprising clock genes and their protein products (e.g., BMAL1, CLOCK, PER1/2/3, CRY1/2, and REV-ERBs), which form interconnected transcriptional-translational feedback loops that control the daily rhythmicity of many other target genes [13]. Disruption of this clockwork and the loss of daily oscillations in immune cells have been associated with reduced protection against infection and accelerated development of metabolic diseases promoted by pro-inflammatory conditions [14, 15].

The circadian system is dominantly entrained by the regular 24-hour cycles of light and dark, resulting from the Earth’s rotation on its axis [16]. Disturbances of these natural light/dark (LD) cycles due to artificial light at night (ALAN) can markedly attenuate daily rhythms of clock and clock-controlled genes in the central oscillator [17] and differentially compromise hormonal, behavioural and metabolic rhythms in the periphery [17, 18]. Furthermore, mistimed circadian rhythms can have adverse effects on physical and mental health, making ALAN an extensively studied global anthropogenic factor to better understand its associated risks and underlying mechanisms [19]. ALAN is a growing phenomenon resulting from excessive and inappropriately timed light exposure, frequently associated with a 24/7 lifestyle. In our recent studies, we demonstrated that low-intensity (dim) ALAN disrupts the daily variability of major WBC populations in rats [20], and this is associated with chronodisruption of the acute inflammatory response to endotoxin, particularly in WBC counts and plasma corticosterone and tumour necrosis factor-α (TNF-α) levels [9].

As the course of inflammation relies on metabolic adaptations to meet energy demands, in the current study we investigated whether the time of day of an immune challenge influences the inflammatory response in metabolic tissues and induces a time-of-day-dependent metabolic response in glucose and lipid pathways at both systemic and molecular levels. To further explore the involvement of circadian control, we hypothesized that acute inflammation-induced metabolic changes might be disrupted by ALAN. Indeed, our previous findings indicated that rats exposed to dim ALAN for 2 weeks showed arrhythmic or phase-advanced expression of key metabolic transcription factors and nutrient sensors in the liver and adipose tissue [18], which may alter their daily sensitivity to inflammatory stimuli.

## Materials and methods

### Animals

Male Wistar rats (Institute of Experimental Pharmacology and Toxicology, Slovak Academy of Sciences, Dobrá Voda, Slovak Republic) were delivered at 16–17 weeks of age (282 ± 4 g body weight). The rats were housed in groups of 3–4 in plastic cages at an ambient temperature of 21.5 ± 1.3 °C and humidity of 55–65%, with food and water available *ad libitum*. During a 2-week acclimation period, the animals were adapted to a standard 12/12-hour LD cycle, with lights on at 06:00 (referred to as Zeitgeber time ZT0). The experimental procedures were approved by the Ethical Committee for the Care and Use of Laboratory Animals at Comenius University in Bratislava, Slovak Republic, and the State Veterinary Authority of the Slovak Republic (Ro-1648/19–221/3).

### Experimental design

**Fig. 1:**
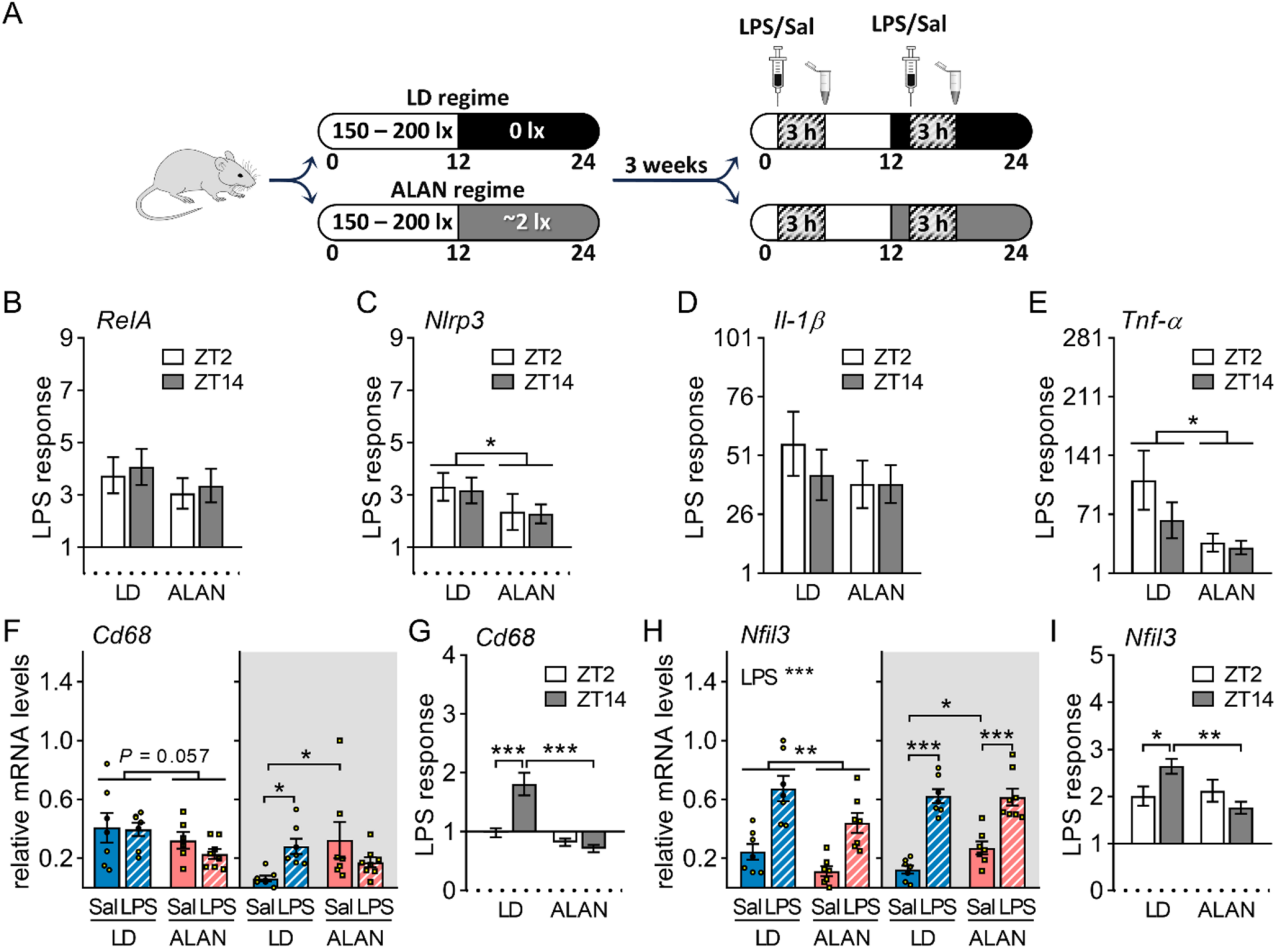
ALAN attenuates hepatic inflammatory response. **A** Outline of the experimental design. Rats were exposed to either the control 12/12 h light/dark regime (LD) or dim ALAN (~ 2 lx) and injected with saline (Sal) or lipopolysaccharide (LPS) at either ZT2 (white background) or ZT14 (shaded background). Zeitgeber time (ZT) 0 = lights on. Data were analysed 3 h post-injection. **B–E** The LPS response (fold change relative to the mean of the saline-injected group) for hepatic *RelA*, *Nlrp3*, *Il-1β* and *Tnf-α* expression. **F–I** Relative mRNA levels and the LPS response for hepatic *Cd68* and *Nfil3*. Bars represent means ± SE (*n* = 6–8 rats per group). Data were evaluated by two-way ANOVA with Bonferroni’s multiple comparison test at *P < 0.05, **P < 0.01 and ***P < 0.001

Following the acclimation period, control rats (LD, *n* = 28) were kept under the standard LD cycle, while ALAN rats (ALAN, *n* = 30) were exposed to dim ALAN (low-intensity light of 2 lx throughout the dark phase). In both lighting conditions, the light phase was illuminated with the broad-spectrum white light of 150–200 lx and a colour temperature of 2900 K. For the ALAN regime, dim light was provided by an LED light strip positioned above the cages, with a colour temperature of 3000 K [18]. After 2 weeks under their respective lighting conditions, the rats were immune-challenged with an i.p. injection of LPS from *Escherichia coli* (serotype 0111:B4; Sigma-Aldrich, St. Louis, MO, USA) at a dose of 1 mg/kg body weight. Half of the rats in each regimen received injections at the beginning of the light phase (ZT2, daytime), while the other half at the beginning of the dark phase (ZT14, nighttime). Unstimulated rats were injected with sterile saline at matching times (Fig. 1A). These times were chosen to cover the peak and trough of the daily rhythm of circulating WBC counts [9].

### Blood and tissue collection

At 24 h after saline or LPS injection, blood samples were collected from a lateral tail vein under isoflurane anaesthesia. Plasma was separated and used for metabolite assays. The rats were allowed to recover for 9 days [9, 21] and then challenged again with LPS following the same design as the first injection. At 3 h post-injection, the rats were sacrificed under brief isoflurane anaesthesia. Blood was collected into empty and ethylenediaminetetraacetic acid-containing tubes to separate serum and plasma by centrifugation at 2500 × g, 15 min, 4 °C. Liver and visceral perirenal white adipose tissue (vWAT) samples were explanted, immediately frozen in liquid nitrogen, and stored at − 80 °C. In the LD regime, the LPS injection and all samplings were performed under dim red light during the dark phase.

### Hormone assays

Plasma leptin and adiponectin concentrations were measured by enzyme-linked immunosorbent assay (ELISA) using commercial kits: Rat Leptin ELISA (RD291001200R; BioVendor, Brno, Czech Republic) and Rat ADP/Acrp30 (Adiponectin) ELISA (ER0006; FineTest, Wuhan, China). Intra-assay variation coefficients were < 8%.

### Biochemical analyses

Plasma/serum glucose, triacylglycerol (TAG), and total cholesterol concentrations were measured using a commercial enzymatic assay (BIO-LA-TEST; Erba Lachema, Brno, Czech Republic), according to the manufacturer’s instructions. The assays were adapted for use in 96-well plates. Plasma free FAs were measured using a colorimetric Free Fatty Acid Assay Kit (ab65341; Abcam, Cambridge, UK).

### RNA isolation and real-time PCR

Liver and vWAT samples were homogenized in QIAzol and TRI Reagent (Molecular Research Center, Cincinnati, OH, USA), respectively, using a FastPrep instrument (MP Biomedicals, Eschwege, Germany), and RNA was isolated with the RNeasy Plus Universal Mini Kit (Qiagen, Hilden, Germany) [18]. The quantity and purity of the isolated RNA were measured on a NanoDrop One spectrophotometer (Thermo Fisher Scientific, Waltham, MA, USA). The Maxima cDNA Synthesis Kit (Thermo Fisher Scientific) and Maxima SYBR Green qPCR Master Mix (Thermo Fisher Scientific) were used for reverse transcription and cDNA amplification, which was performed on a CFX Connect real-time PCR detection system (Bio-Rad, Hercules, CA, USA). Relative RNA expression was calculated using the standard curve method. In liver, the expression of target genes was normalized to the geometric mean of β-actin and ribosomal protein S29 (*Rps29*), and in vWAT to *Rps29* expression. Primer sequences for the housekeeping and target genes are listed in Supplementary Table S1.

### Western blot

Liver samples were homogenized on ice in sucrose buffer with protease inhibitors and processed according to a previously published protocol [9, 20]. Protein concentrations were measured using the Pierce BCA assay kit (Thermo Fisher Scientific). Proteins (30–50 µg) were loaded onto SDS-PAGE gel and transferred to a 0.45-nm nitrocellulose membrane. The membranes were blocked with 5% (non-fat) milk in Tris-buffered saline (TBS) for 2 × 30 min at room temperature. After blocking, the membranes were incubated with primary antibodies diluted in 1% milk in TBS for 18 h at 4 °C. The primary antibodies used were rabbit anti-BMAL1 (ab3350; Abcam; 1:700), rabbit anti-phosphorylated-NF-κB/p65 (Ser536) (ab76302; Abcam; 1:1000) and rabbit anti-NF-κB/p65 (ab16502; Abcam; 1:1000). The membranes were washed in TBS and incubated with anti-rabbit horseradish peroxidase-conjugated secondary antibody (7074; Cell Signaling Technology, Danvers, MA, USA; 1:2000, in 1% milk in TBS) for 1 h at room temperature. All membranes were reprobed with mouse anti-glyceraldehyde-3-phosphate dehydrogenase (GAPDH) antibody (MAB374; Sigma-Aldrich; 1:7000) and an anti-mouse secondary antibody (7076; Cell Signaling Technology; 1:2000). The signal was visualized and detected using Clarity Western ECL substrate (1705061; Bio-Rad) and the Vü-C chemiluminescence imaging system (Pop-Bio Imaging, Cambridge, UK). The membranes were analysed with Image Studio Lite software (LI-COR Biosciences, Lincoln, NE, USA). The signal for all proteins was normalized to GAPDH and presented as a relative quantity.

### Statistical analysis

Statistical analysis was performed using GraphPad Prism 8 (GraphPad Software, San Diego, CA, USA). The sample size was estimated to be between 6 and 8 animals per group based on our previous animal studies and the recommended calculation method [22]. Data were analysed by two-way analysis of variance (ANOVA) with Bonferroni’s multiple comparison test. If the data did not pass the Shapiro Wilk test for a normal distribution, logarithmic or square root-transformed data were used. The LPS response was calculated as the fold change of LPS-stimulated values relative to the mean of the saline-injected group. Data are presented as means ± standard error (SE).

## Results

### ALAN attenuates hepatic and adipose inflammatory response to LPS

In the liver, mRNA levels of NF-κB subunit *p65/RelA*, NLR family pyrin domain containing 3 (*NLRP3*), interleukin-1β (*IL-1β*), and *Tnf-α* were upregulated 3 h after LPS injection (*P* < 0.001 for all), regardless of the time of stimulation (Fig. 1B–E, S1A–D). Notably, ALAN rats showed a reduced LPS response in *Nlrp3* (*P* < 0.05; Fig. 1C) and *Tnf-α* expression (*P* < 0.05; Fig. 1E). Total p65 protein levels were unaffected by LPS in the liver (Fig. S1E, F). However, ALAN rats displayed higher daytime levels of phosphorylated p65 (Pp65) than LD rats (*P* < 0.01), and these levels decreased after LPS injection (*P* < 0.01), whereas no response occurred in the LD regime (Fig. S1E). Similar changes were observed in the hepatic Pp65/p65 ratio (Fig. S1E). Next, the expression of a macrophage marker, CD68, was upregulated only after nighttime LPS challenge in LD rats (*P* < 0.05; Fig. 1F, G), whereas no response was noted in ALAN rats. On the other hand, ALAN increased nighttime *Cd68* expression (*P* < 0.05; Fig. 1F) and tended to reduce daytime levels compared with the LD regime (*P* = 0.057; Fig. 1F). In both LD and ALAN rats, the LPS challenge upregulated nuclear factor, interleukin-3 regulated (NFIL3) (*P* < 0.001; Fig. 1H), a transcription factor that integrates immune, circadian, and metabolic processes. In the LD regime, the hepatic *Nfil3* response was higher after nighttime than daytime LPS stimulation (*P* < 0.05), whereas this day–night difference was suppressed by ALAN (Fig. 1I). Additionally, ALAN rats had lower daytime (*P* < 0.01) and higher nighttime (*P* < 0.05) *Nfil3* mRNA levels than LD rats (Fig. 1H), indicating a disrupted daily rhythm.

**Fig. 2:**
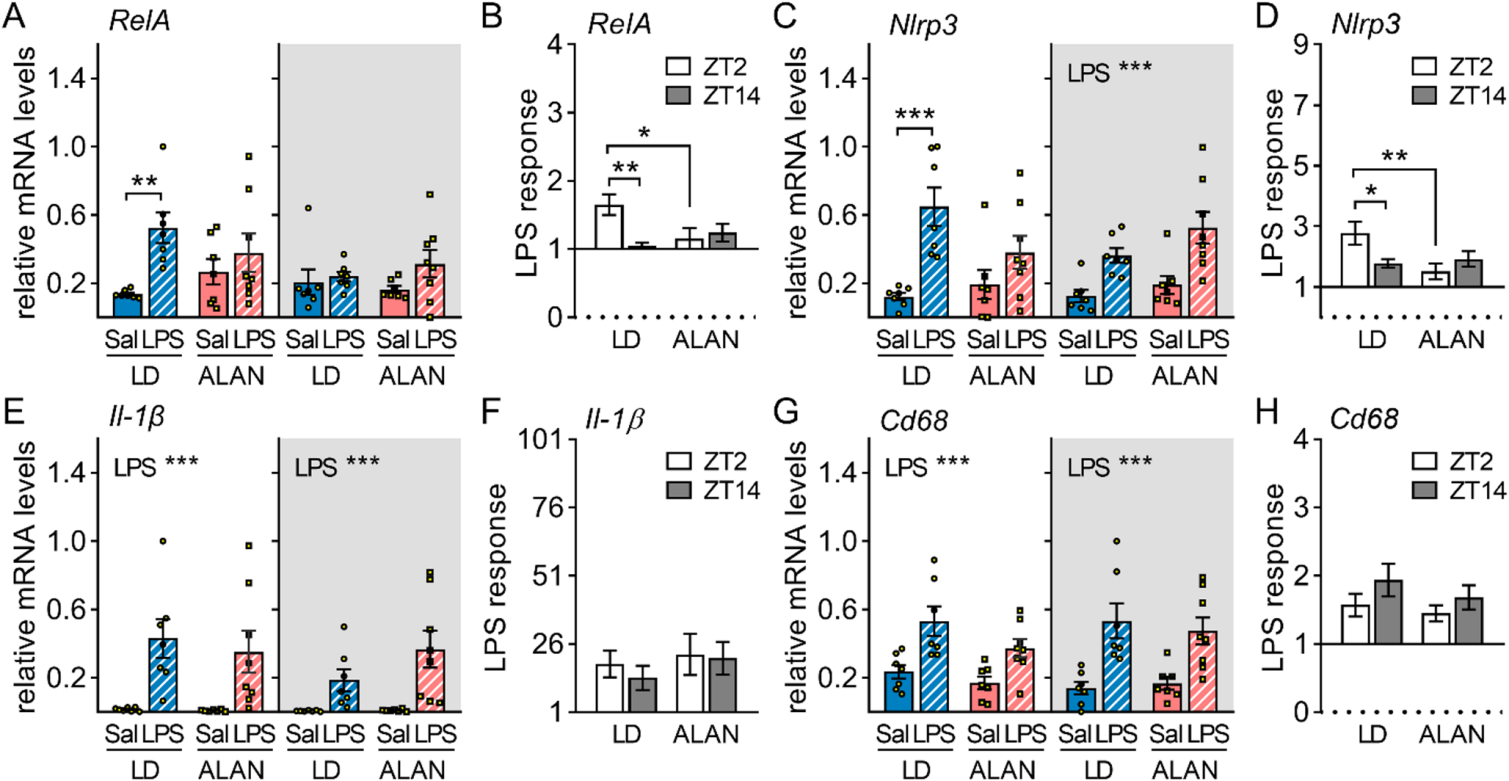
ALAN suppresses adipose inflammatory response to daytime lipopolysaccharide (LPS) injection. Rats were exposed to either the control 12/12 h light/dark regime (LD) or dim ALAN (~ 2 lx) and injected with saline (Sal) or LPS at either ZT2 (white background) or ZT14 (shaded background). Zeitgeber time (ZT) 0 = lights on. Data were analysed 3 h post-injection. Relative mRNA levels and the LPS response (fold change relative to the mean of the saline-injected group) for *RelA* (**A**,** B**), *Nlrp3* (**C**,** D**), *Il-1β* (**E**,** F**) and *Cd68* (**G**,** H**). Bars represent means ± SE (*n* = 7–8 rats per group). Data were evaluated by two-way ANOVA with Bonferroni’s multiple comparison test at *P < 0.05, **P < 0.01 and ***P < 0.001

In vWAT, daytime LPS injection upregulated *RelA* mRNA levels in LD rats (*P* < 0.01), while ALAN abolished this response (Fig. 2A). Conversely, nighttime LPS stimulation did not induce *RelA* expression in either regime (Fig. 2A), underlying a time-of-day-dependent response present only in LD rats (*P* < 0.01; Fig. 2B). Adipose *Nlrp3* mRNA levels were upregulated after both daytime (*P* < 0.001) and nighttime LPS injections (*P* < 0.001) in the LD regime (Fig. 2C), but ALAN suppressed the daytime LPS-induced increase in *Nlrp3* (Fig. 2C), thereby eliminating the day–night differences observed in LD rats (*P* < 0.05; Fig. 2D). LPS injection also upregulated *Il-1β* (*P* < 0.001; Fig. 2E, F) and *Cd68* mRNA levels (*P* < 0.001; Fig. 2G, H) in vWAT, but no effects of treatment time or lighting regime were observed.

### Glucose metabolism in the liver is more responsive to daytime than nighttime LPS

**Fig. 3:**
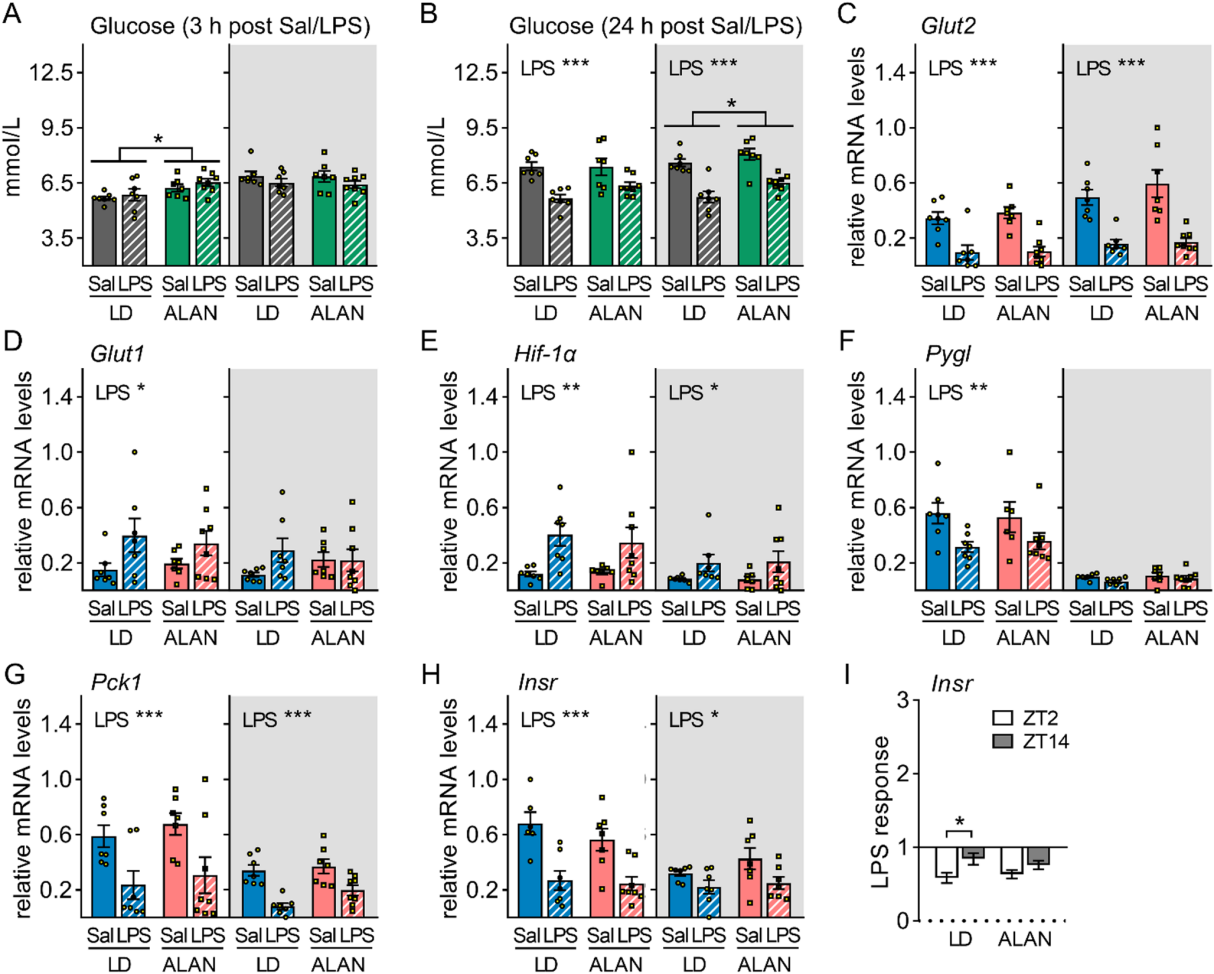
Glucose metabolism in the liver is more responsive to daytime than nighttime lipopolysaccharide (LPS). Rats were exposed to either the control 12/12 h light/dark regime (LD) or dim ALAN (~ 2 lx) and injected with saline (Sal) or LPS at either ZT2 (white background) or ZT14 (shaded background). Zeitgeber time (ZT) 0 = lights on. Plasma glucose levels measured 3 h (**A**) and 24 h (**B**) post-injection. **C–G** Relative mRNA levels of hepatic glucose transporters *Glut2* and *Glut1*, hypoxia-inducible factor-1α (*Hif-1α*), glycogen phosphorylase L (*Pygl*) and phosphoenolpyruvate carboxykinase 1 (*Pck1*) in the liver 3 h post-injection. **H**,** I** Relative mRNA levels and the LPS response (fold change relative to the mean of the saline-injected group) for insulin receptor (*Insr*). Bars represent means ± SE (*n* = 6–8 rats per group). Data were evaluated by two-way ANOVA with Bonferroni’s multiple comparison test at *P < 0.05, **P < 0.01 and ***P < 0.001

In response to LPS, glucose levels exhibit dynamic changes manifested by initial hyperglycaemia followed by hypoglycaemia in the later phases of inflammation [23], reflecting progressive shifts in the balance between glucose production and utilization. We observed that plasma glucose levels were not affected 3 h after LPS injection (Fig. 3A) but decreased 24 h after stimulation, regardless of treatment time or ALAN exposure (*P* < 0.001; Fig. 3B). Notably, ALAN rats had higher glucose levels than LD rats (ZT2: *P* < 0.05; ZT14: *P* < 0.05; Fig. 3A, B). Both daytime and nighttime LPS challenges acutely reduced expression of the major glucose transporter (GLUT2) in the liver (*P* < 0.001; Fig. 3C). By contrast, *Glut1* was upregulated after daytime LPS injection (*P* < 0.05; Fig. 3D), whereas nighttime immune stimulation had no significant effect, showing a time-of-day-dependent response. These LPS-induced changes in glucose transporters were not influenced by ALAN (Fig. 3C, D). Consistently, expression of hypoxia-inducible factor-1-alpha, which transactivates *Glut1* and glycolytic enzymes, was upregulated by LPS challenge (ZT2: *P* < 0.01; ZT14: *P* < 0.05; Fig. 3E), although no time-of-day-dependent differences and no ALAN effects were observed. Interestingly, expression of glycogen phosphorylase L (PYGL), a rate-limiting enzyme in glycogen breakdown, was reduced only after daytime LPS injection (*P* < 0.01; Fig. 3F), regardless of the lighting regime. Next, representatives of the gluconeogenic pathway, namely phosphoenolpyruvate carboxykinase 1 (PCK1) and the forkhead box O1 transcription factor (FOXO1), were acutely downregulated at the mRNA levels after both daytime and nighttime LPS challenges in both LD and ALAN rats (*Pck1*: *P* < 0.001; *Foxo1*: *P* < 0.001; Fig. 3G, S2A). Similarly, expression of insulin receptor (INSR) decreased after LPS (ZT2: *P* < 0.001; ZT14: *P* < 0.05; Fig. 3H) but the response was greater after daytime than nighttime stimulation in LD rats (*P* < 0.05; Fig. 3I), suggesting a time-of-day-dependent effect of inflammation on insulin sensitivity. Importantly, day–night variability was suppressed in ALAN rats.

### ALAN impairs daily variability in lipid metabolic response to LPS in the liver

**Fig. 4:**
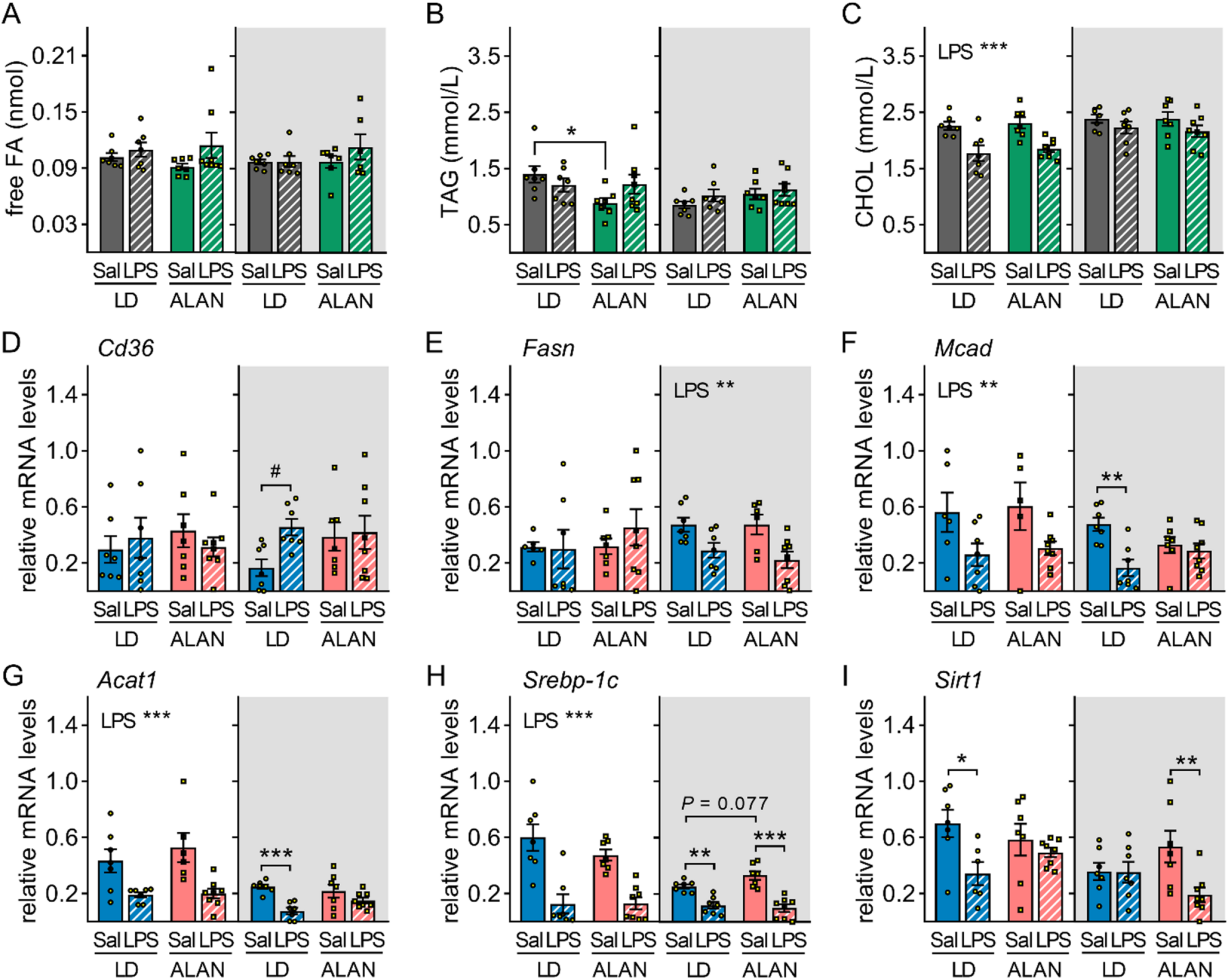
ALAN impairs daily variability in lipid metabolic response to lipopolysaccharide (LPS) in the liver. Rats were exposed to either the control 12/12 h light/dark regime (LD) or dim ALAN (~ 2 lx) and injected with saline (Sal) or LPS at either ZT2 (white background) or ZT14 (shaded background). Zeitgeber time (ZT) 0 = lights on. Data were analysed 3 h post-injection. **A–C** Free fatty acid (FA), triacylglycerol (TAG) and total cholesterol (CHOL) plasma levels. **D–I** Relative mRNA levels of hepatic *Cd36*, fatty acid synthase (*Fasn*), medium-chain acyl-CoA dehydrogenase (*Mcad*), acetyl-CoA acetyltransferase 1 (*Acat1*), sterol regulatory element binding protein-1c (*Srebp-1c*) and sirtuin 1 (*Sirt1*). Bars represent means ± SE (*n* = 6–8 rats per group). Data were evaluated by two-way ANOVA with Bonferroni’s multiple comparison test at *P < 0.05, **P < 0.01, ***P < 0.001 and ^#^*P* = 0.071

Acute inflammation induces significant changes in lipid metabolism. We observed no effects on plasma free FA and TAG levels either 3–24 h after LPS injection, regardless of treatment time (Fig. 4A, B, S2B). In contrast, plasma cholesterol concentrations decreased 3 h after daytime LPS injection (*P* < 0.001; Fig. 4C) and increased 24 h after nighttime stimulation in both LD and ALAN rats (*P* < 0.001; Fig. S2C). Interestingly, ALAN lowered daytime TAG levels compared to the LD regime (*P* < 0.05; Fig. 4B), suggesting a disturbed daily TAG rhythm. In LD rats, hepatic gene expression of the FA translocase (CD36) did not change after daytime LPS injection but tended to increase after nighttime stimulation (*P* = 0.071; Fig. 4D). This response was absent in the ALAN regime. In both LD and ALAN rats, mRNA levels of FA synthase (FASN) were downregulated only after nighttime LPS challenge (*P* < 0.01) and remained unchanged following daytime stimulation (Fig. 4E). Daytime and nighttime LPS injections acutely downregulated the expression of mitochondrial enzymes, medium-chain acyl-CoA dehydrogenase (MCAD) (ZT2: *P* < 0.01; ZT14: *P* < 0.01; Fig. 4F) and acetyl-CoA acetyltransferase 1 (ACAT1) (ZT2: *P* < 0.001; ZT14: *P* < 0.001; Fig. 4G) in LD rats, suggesting reduced FA oxidation and ketone body production in the liver. Interestingly, ALAN suppressed the LPS-induced decrease in *Mcad* and *Acat1* expression specifically after nighttime stimulation (Fig. 4F, G).

Next, we analysed hepatic expression of several metabolic sensors that can mediate changes in lipid metabolism during acute inflammation. The transcription factor PPARα was downregulated at the mRNA level after both daytime (*P* < 0.001) and nighttime (*P* < 0.001; Fig. S2D) LPS injections, irrespective of the ALAN regime. Expression of another transcription factor, SREBP-1c, was also reduced after LPS (ZT2: *P* < 0.001; ZT14: *P* < 0.01 in LD and *P* < 0.001 in ALAN; Fig. 4H), showing a greater response to daytime than nighttime stimulation in the LD regime (*P* < 0.01; Fig. S2E). ALAN rats showed a trend toward higher *Srebp-1c* expression during the night phase than LD rats (*P* = 0.077) and exhibited no day–night difference in the LPS response (Fig. 4H, S2E). Hepatic expression of the NAD^+^-dependent deacetylase, sirtuin 1 (SIRT1), was downregulated following LPS challenge, but this response was time- and lighting regime-specific (Fig. 4I). In LD rats, *Sirt1* decreased after daytime stimulation (*P* < 0.05), while in ALAN rats, it decreased after nighttime stimulation (*P* < 0.01).

### ALAN enhances anabolic and suppresses catabolic response to LPS in vWAT

**Fig. 5:**
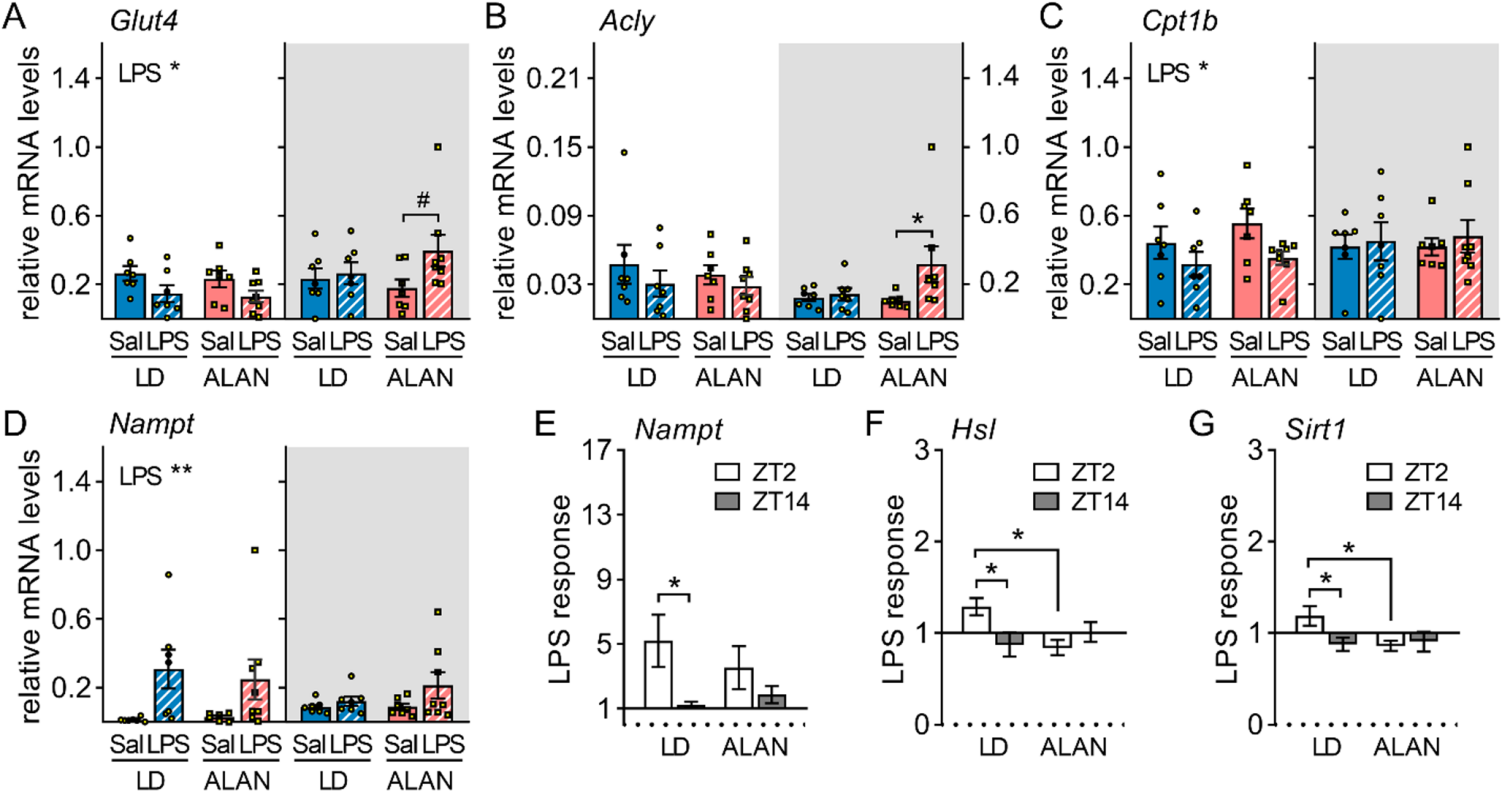
ALAN enhances anabolic and suppresses catabolic response to lipopolysaccharide (LPS) in visceral fat (vWAT). Rats were exposed to either the control 12/12 h light/dark regime (LD) or dim ALAN (~ 2 lx) and injected with saline (Sal) or LPS at either ZT2 (white background) or ZT14 (shaded background). Zeitgeber time (ZT) 0 = lights on. Data were analysed 3 h post-injection. **A–D** Relative mRNA levels for glucose transporter *Glut4*, ATP citrate lyase (*Acly*), carnitine palmitoyltransferase 1b (*Cpt1b*) and nicotinamide phosphoribosyltransferase (*Nampt*). **E–G** The LPS response (fold change relative to the mean of the saline-injected group) for *Nampt*, hormone-sensitive lipase (*Hsl*) and sirtuin 1 (*Sirt1*). Bars represent means ± SE (*n* = 6–8 rats per group). Data were evaluated by two-way ANOVA with Bonferroni’s multiple comparison test at *P < 0.05, **P < 0.01 and ^#^*P* = 0.066

Adipose tissue is important for linking metabolic and immune responses during inflammation [24]. In vWAT, daytime LPS injection reduced the expression of the insulin-regulated glucose transporter 4 (GLUT4) regardless of ALAN (*P* < 0.05; Fig. 5A). Nighttime LPS stimulation did not induce a *Glut4* response in LD rats but tended to upregulate *Glut4* in ALAN rats (*P* = 0.066; Fig. 5A). Nighttime LPS administration also upregulated ATP citrate lyase (ACLY), an enzyme involved in FA synthesis, specifically under the ALAN regime (*P* < 0.05; Fig. 5B), with no *Acly* response observed after daytime injection. Carnitine palmitoyltransferase 1b (CPT1b) is a key enzyme involved in the transport of long-chain FAs into the mitochondria for oxidation. Adipose *Cpt1b* expression was downregulated following daytime LPS injection (*P* < 0.05; Fig. 5C), whereas nighttime stimulation did not elicit a *Cpt1b* response, indicating a time-of-day-dependent effect on FA oxidation in vWAT. Adipose expression of nicotinamide phosphoribosyltransferase (NAMPT), a rate-limiting enzyme in the NAD^+^ salvage pathway, was elevated only after daytime LPS stimulation (*P* < 0.01; Fig. 5D). Although the upregulation was observed in both LD and ALAN rats, ALAN suppressed the daily variability in this LPS response (Fig. 5E).

A representative enzyme of TAG catabolism in adipose tissue is hormone-sensitive lipase (HSL). The expression of *Hsl* was influenced by an interaction between daytime LPS challenge and ALAN (*P* = 0.072; Fig. S3A), manifested by an upregulation after daytime compared to nighttime stimulation in LD rats (*P* < 0.05) and an opposite daytime response in ALAN rats (*P* < 0.05; Fig. 5F). The same pattern of LPS response was observed for *Sirt1* (Fig. 5G, S3B). No effects of LPS or ALAN were observed on the expression of *Cd36* and lipoprotein lipase (Fig. S3C, D), which are involved in FA uptake in adipose tissue. However, adipose expression of PPARγ, an important transcriptional regulator of lipid metabolism in WAT, was downregulated following LPS injection, with treatment time and ALAN showing no effect on this response (Fig. S3E).

### ALAN attenuates adipokine response to LPS

**Fig. 6:**
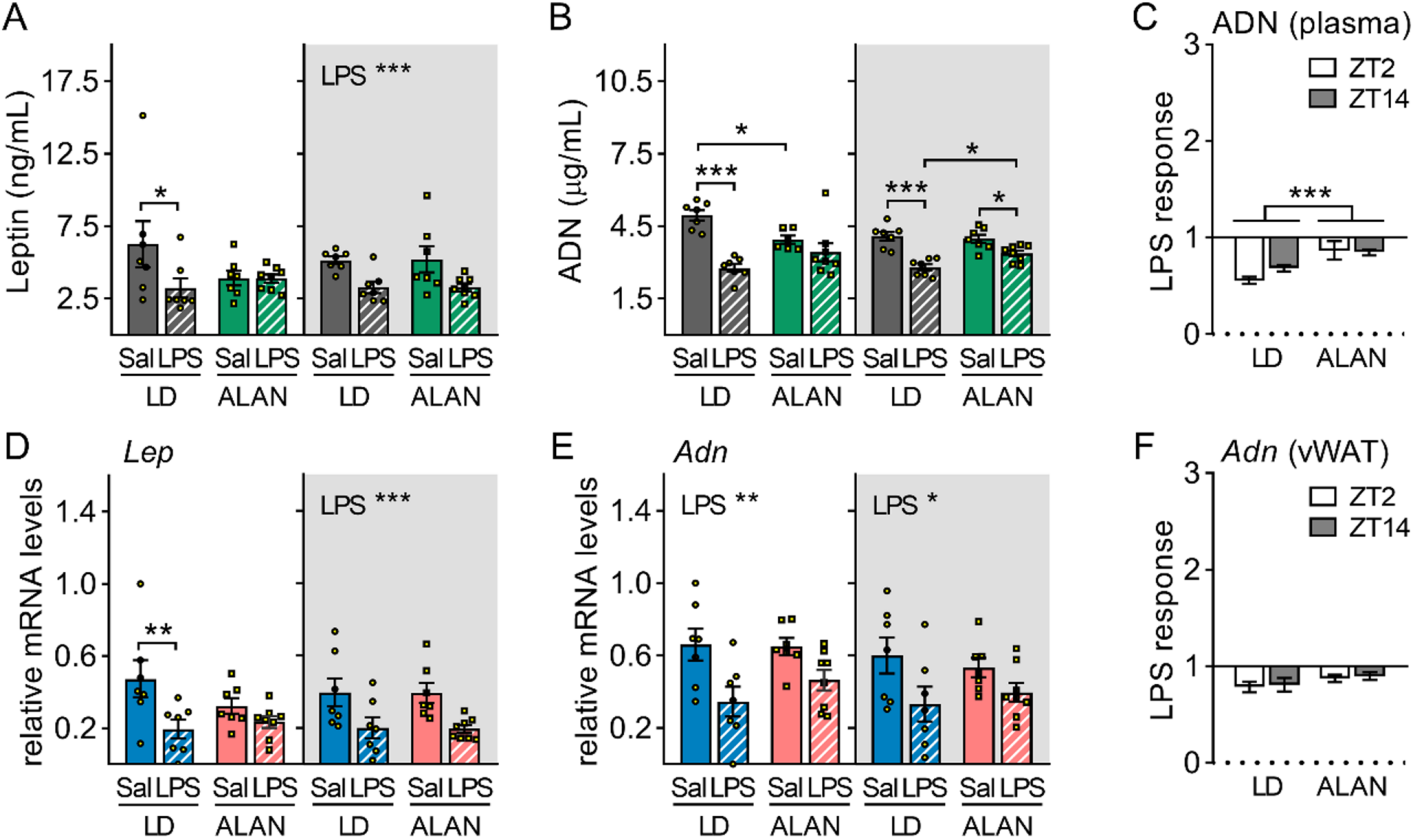
ALAN attenuates adipokine response to lipopolysaccharide (LPS). Rats were exposed to either the control 12/12 h light/dark regime (LD) or dim ALAN (~ 2 lx) and injected with saline (Sal) or LPS at either ZT2 (white background) or ZT14 (shaded background). Zeitgeber time (ZT) 0 = lights on. Blood and visceral fat (vWAT) samples were collected 3 h post-injection. **A**,** B** Plasma levels of leptin (LEP) and adiponectin (ADN). **C** The LPS response (fold change relative to the mean of the saline-injected group) for plasma ADN. Relative mRNA levels of *Lep* (**D**) and *Adn* (**E**) and the LPS response for *Adn* (**F**) in vWAT. Bars represent means ± SE (*n* = 6–8 rats per group). Data were evaluated by two-way ANOVA with Bonferroni’s multiple comparison test at *P < 0.05, **P < 0.01 and ***P < 0.001

Adipokines released into the circulation, such as leptin and adiponectin, are key mediators of metabolic crosstalk between adipose tissue and the liver [25]. Plasma leptin and adiponectin levels decreased 3 h after daytime LPS injection in the LD regime (leptin: *P* < 0.05; adiponectin: *P* < 0.001), whereas no response was observed under ALAN (Fig. 6A, B). However, unstimulated ALAN rats had lower daytime adiponectin levels than LD rats (*P* < 0.05; Fig. 6B). Nighttime LPS stimulation reduced plasma levels of both adipokines regardless of the lighting regime (leptin: *P* < 0.001; adiponectin: *P* < 0.001 in LD and *P* < 0.05 in ALAN group), but post-stimulation adiponectin levels were higher in ALAN than in LD rats (*P* < 0.05; Fig. 6B), indicating an attenuated response to LPS (*P* < 0.001; Fig. 6C). Leptin gene expression in vWAT closely mirrored LPS-induced changes observed in plasma (Fig. 6A, D). Specifically, ALAN prevented the decrease in leptin mRNA levels after daytime LPS injection (Fig. 6D). Nighttime LPS injection reduced leptin (*P* < 0.001) and adiponectin mRNA levels (*P* < 0.05) in both LD and ALAN rats (Fig. 6D, E), but ALAN tended to attenuate the adiponectin response to LPS (*P* = 0.091; Fig. 6F).

### Liver and adipose molecular clocks show time-of-day-dependent response to LPS

**Fig. 7:**
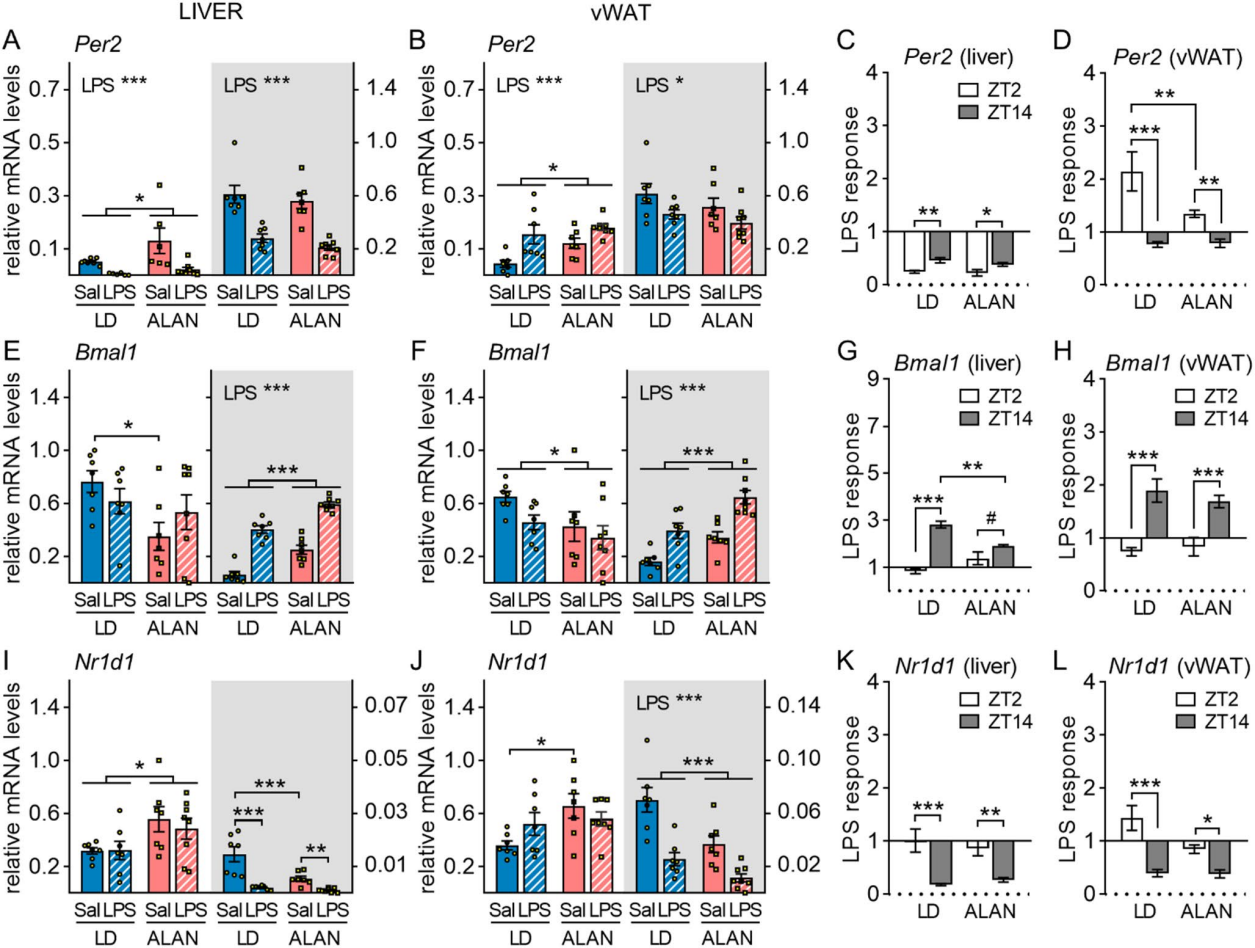
Liver and adipose molecular clocks show time-of-day-dependent response to LPS. Rats were exposed to either the control 12/12 h light/dark regime (LD) or dim ALAN (~ 2 lx) and injected with saline (Sal) or lipopolysaccharide (LPS) at either ZT2 (white background) or ZT14 (shaded background). Zeitgeber time (ZT) 0 = lights on. Relative mRNA levels of clock components *Per2* (**A**,** B**), *Bmal1* (**E**,** F**) and *Nr1d1* (*Rev-erbα*) (**I**,** J**) in the liver and visceral fat (vWAT) 3 h post-injection. The LPS response (fold change relative to the mean of the saline-injected group) for *Per2* (**C**,** D**), *Bmal1* (**G**,** H**) and *Nr1d1* (**K**,** L**). Bars represent means ± SE (*n* = 6–8 rats per group). Data were evaluated by two-way ANOVA with Bonferroni’s multiple comparison test at *P < 0.05, **P < 0.01, ***P < 0.001 and ^#^*P* = 0.067

Hepatic expression of the core clock gene *Per2* was downregulated 3 h after LPS stimulation (*P* < 0.001; Fig. 7A), regardless of the time of injection, but showed higher decrease after daytime than nighttime immune stimulation in both LD (*P* < 0.01) and ALAN rats (*P* < 0.05; Fig. 7C). Interestingly, *Per2* expression in vWAT increased in response to daytime LPS (*P* < 0.001) and decreased after nighttime LPS challenge (*P* < 0.05; Fig. 7B). Gene expression of the other molecular clock components *Bmal1*, *Nr1d1* (*Rev-erbα*) and D-box binding transcription factor (*Dbp*) did not change in response to daytime LPS injection in either the liver or vWAT (Fig. 7E–L, S4A, B). However, nighttime LPS stimulation upregulated *Bmal1* (*P* < 0.001; Fig. 7E–H) and downregulated both *Nr1d1* (*P* < 0.001; Fig. 7I–L) and *Dbp* (*P* < 0.001; Fig. S4A, B). This time-of-day-dependent response to LPS was further confirmed by BMAL1 protein expression in the liver (Fig. S4C). ALAN reduced the LPS response in adipose *Per2* (*P* < 0.01; Fig. 7D) and hepatic *Bmal1* expression compared to the LD regime (*P* < 0.01; Fig. 7G). Furthermore, irrespective of immune stimulation, ALAN affected clock gene expression in a phase-dependent manner, with lower daytime expression of *Bmal1* (Fig. 7E, F) and higher daytime expression of *Per2* (Fig. 7A, B), *Nr1d1* (Fig. 7I, J) and *Dbp* (Fig. S4A, B) in ALAN than LD rats, while opposite between-group differences were observed for nighttime clock gene expression of *Bmal1*, *Nr1d1* and *Dbp*.

**Fig. 8:**
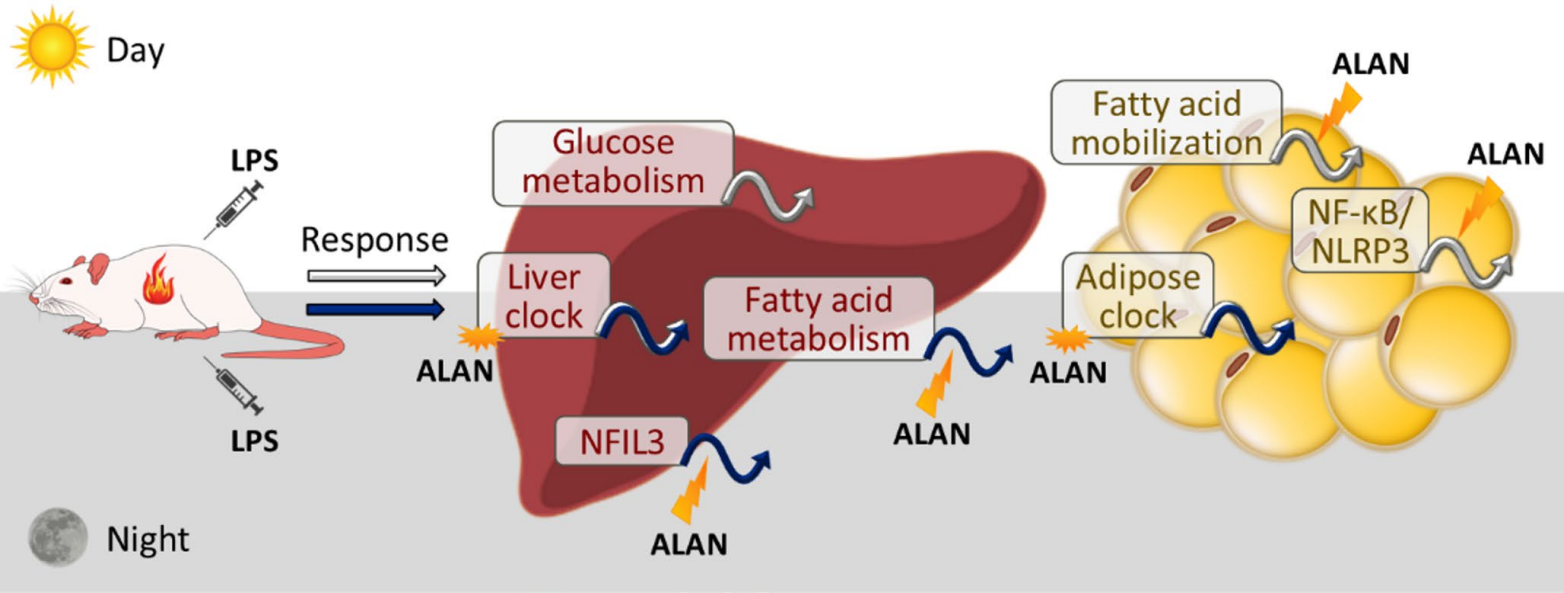
Illustrated summary of the results. Under a regular light/dark cycle, time-of-day-dependent lipopolysaccharide (LPS) challenge triggers distinct metabolic and inflammatory responses in rat liver and adipose tissue. Daytime LPS injection induced a greater response in hepatic glucose metabolism and adipose fatty acid mobilization, which was associated with daily variability in nuclear factor-kappa B (NF-κB)/NLR family, pyrin domain containing 3 (NLRP3) inflammasome pathway. Fatty acid metabolism in the liver was more responsive to nighttime LPS injection, which also induced a greater response in the nuclear factor interleukin-3 regulated (NFIL3). In addition, the peripheral metabolic clocks exhibited a time-of-day-specific response to LPS that varied according to the clock gene and tissue type. Disruption of the light-dark cycle by dim artificial light at night (ALAN) disturbed liver and adipose circadian clocks and impaired lipid metabolic adaptations induced by acute inflammation

## Discussion

Systemic and cellular metabolic adaptations during inflammation and infection play a substantial role in shaping immune defence outcomes. In this study, we focused on the inflammatory and metabolic responses to a time-of-day-dependent LPS challenge in rat liver and adipose tissue under regular and disturbed light cycles to examine daily variability in inflammation-induced metabolic changes and the involvement of the circadian clock (Fig. 8). Our results revealed that a daytime LPS challenge elicited greater *RelA*/*Nlrp3* upregulation in vWAT than the nighttime stimulation, while no time-specific response in this inflammatory pathway was observed in the liver. Nevertheless, hepatic glucose metabolism was more responsive to daytime than nighttime endotoxin, whereas the opposite trend was observed for FA uptake and synthesis. In vWAT, higher FA mobilization was triggered by daytime than nighttime LPS stimulation, corresponding to daily variation in the adipose inflammatory response. A link between the circadian clock and LPS-evoked metabolic changes was demonstrated at several levels. In both liver and adipose tissue, the LPS response in clock gene expression varied according to the time of immune stimulation. Previous research has shown that dim ALAN can disrupt circadian rhythms in metabolism and immunity [9, 18], thereby compromising their predictive value in response to challenging stimuli. Indeed, our current findings demonstrate that ALAN attenuates the hepatic inflammatory response and impairs daily variability in FA metabolism adaptation following LPS in the liver. The molecular mechanism may also involve the transcription factor NFIL3, as ALAN abolished the time-of-day-dependent response in hepatic *Nfil3* upregulation after LPS stimulation. Moreover, ALAN suppressed the daytime responsiveness of adipose tissue to endotoxin, as evidenced by suppressed adipokine, catabolic and inflammatory responses. Conversely, ALAN enhanced the nighttime LPS-induced anabolic response in vWAT.

We observed increased plasma glucose levels in ALAN rats, which is consistent with our previous findings [26] and may be attributed to impaired daily rhythms of plasma glucose and food intake in the ALAN regime [17, 18] or to direct light-induced hyperglycaemia [27]. Our data confirm a hypoglycaemic response 24 h after LPS, with no variation based on the timing of immune stimulation. Interestingly, glucose transporters were affected 3 h post-LPS injection in a time-of-day-dependent manner. Hepatic *Glut1* expression increased and adipose *Glut4* expression decreased only after daytime LPS, suggesting that endotoxin-related changes in glucose transport involve circadian regulation. In the liver, endotoxemia has been shown to markedly increase GLUT1 protein in both Kupffer and hepatic parenchymal cells, supporting glucose uptake and glucose output, respectively [28]. Thus, we hypothesize that the LPS-induced increase in *Glut1* expression during the light phase promotes hepatic glucose flux when blood glucose levels are at their daily minimum and endogenous glucose production is reduced. Indeed, our data show decreased expression of gluconeogenic genes in response to endotoxin. Moreover, *Pygl* expression decreased specifically after daytime LPS, indicating a time-dependent reduction in liver glycogen mobilization. Interestingly, another study in rats showed the time-of-day-dependent sensitivity of the liver-spinal axis to LPS stimulation that can contribute to the daily fluctuations of the inflammatory response [29].

Acute inflammation induces significant changes in lipid metabolism, including cholesterol levels [6]. In Syrian hamsters, LPS elevated serum cholesterol concentrations 16 h after stimulation by increasing cholesterol biosynthesis in the liver [30]. In our study, LPS-induced changes in cholesterol levels varied over 24 h and according to the timing of immune stimulation, showing a decrease 3 h after daytime LPS and an increase 24 h after nighttime challenge. Early hypocholesterolaemia following LPS may result from increased cholesterol uptake by the adrenals for corticosteroid biosynthesis [31]. Several studies have found a greater corticosterone response to daytime than nighttime LPS [9, 23], which corresponds with the daytime-specific cholesterol reduction observed early after LPS stimulation. Although ALAN did not affect this cholesterol response to LPS, we previously reported that daily rhythms of cholesterol and TAG levels were eliminated in ALAN rats [18]. Consistently, our current data showed phase-dependent differences in TAG levels between LD and ALAN rats, providing further evidence of disturbed metabolite rhythms under the ALAN regime.

In the liver, most of the metabolic genes involved in lipid pathways were acutely downregulated by the LPS challenge, indicating suppression of FA synthesis, FA oxidation, and ketogenesis. The decrease in *Fasn* expression was dependent on the timing of LPS treatment, occurring only after nighttime stimulation. Hepatic *Fasn* expression, along with other genes responsible for de novo FA synthesis, exhibits a daily rhythm peaking during the dark phase [32]. Thus, the endotoxin-induced acute suppression of *Fasn* may correspond to the daily phase of maximal FA synthesis. In contrast to *Fasn*, hepatic *Cd36* expression tended to be upregulated specifically after nighttime LPS, indicating an increased FA uptake in the liver during the dark phase. This response was not observed in ALAN rats, potentially because of already elevated *Cd36* levels as shown in our previous study [26]. Moreover, CD36 functions as a scavenger receptor that can promote an LPS-induced proinflammatory phenotype in macrophages [33]. We observed that the *Cd36* response to LPS corresponded to the time-of-day-dependent response of hepatic *Cd68* expression, reflecting daily variability in liver macrophage activation, which was absent in ALAN rats. During inflammation, hepatic FAs are preferentially used for re-esterification and TAG synthesis, which is associated with reduced FA oxidation and ketogenesis [6]. Here, we found that ALAN suppressed the LPS-induced inhibition of hepatic *Mcad* and *Acat1* expression specifically after nighttime stimulation, thereby disrupting the repressive response in FA oxidation and ketogenesis in a time-of-day-dependent manner.

Acute inflammation has been shown to downregulate hepatic expression of several metabolic sensors [7] that mediate metabolic changes during the immune challenges. Consistently, we observed LPS-induced downregulation of *Pparα* and *Srebp-1c*, which are important hepatic regulators of catabolic and lipogenic pathways, respectively. While the *Pparα* response showed no day–night differences, a greater decrease in *Srebp-1c* was observed after daytime than nighttime stimulation in the LD regime. ALAN eliminated this time-of-day-dependent variation in response to LPS, likely by disrupting the daily rhythm of *Srebp-1c* expression [18]. Insulin signalling has been shown to participate in the transcriptional control of *Srebp-1c* in the liver [34]. Like the *Srebp-1c* response, a greater downregulation of hepatic *Insr* expression was observed after daytime than nighttime LPS in the LD regime, suggesting a time-of-day-dependent effect of inflammation on insulin sensitivity. This day–night difference was absent in ALAN rats, confirming that ALAN abolishes circadian control.

Another key energy sensor is SIRT1, which modulates the activity and expression of many metabolic transcription factors, including core clock components, thereby integrating energy homeostasis with circadian control of metabolic processes [35]. Our data showed reduced hepatic *Sirt1* expression specifically after daytime LPS stimulation in the LD regime, suggesting a role for circadian regulation. Interestingly, ALAN shifted this response to the opposite phase, as *Sirt1* expression in ALAN rats decreased only after nighttime LPS. This altered *Sirt1* responsiveness to endotoxin may stem from the loss of daily rhythmicity in *Sirt1* expression, which we recently found in the liver and adipose tissue of ALAN rats [18]. SIRT1 has been shown to control the metabolic switch from glycolysis to FA oxidation between the early and late stages of acute inflammation [36]. Thus, an inverse time-of-day-dependent *Sirt1* responsiveness to LPS may interfere with the ability of ALAN rats to maintain metabolic adaptations during inflammation.

Distinct metabolic responses specific to the time of day of endotoxin treatment suggest regulation by the circadian clock. Core clock genes indeed respond to inflammatory stimuli in both the suprachiasmatic nucleus and peripheral tissues [11, 37, 38]. Recently, we found that this response varies with the timing of LPS stimulation in the rat kidney [9]. Likewise, our current data show that the hepatic and adipose molecular clocks are responsive to LPS in a time-of-day-dependent manner. The core clock gene *Per2* was downregulated in the liver and upregulated in vWAT after daytime LPS injection. Conversely, the expression of clock components *Bmal1*, *Rev-erbα* and *Dbp* was more responsive to nighttime than daytime endotoxin challenge. Together, these results demonstrate a direct link between LPS-mediated metabolic changes and the circadian clock. This link may be further supported by the time-of-day-dependent response in hepatic *Nfil3* expression, a transcription factor that integrates circadian, immune, and metabolic regulation. For instance, the phase of NFIL3 circadian oscillations can determine the magnitude of the inflammatory response in macrophages [39], and NFIL3 in the small intestine has been shown to promote the expression of *Cd36* and other genes involved in lipid uptake and metabolism [40]. Importantly, we found that ALAN attenuated the nighttime LPS-induced upregulation of *Nfil3* and *Bmal1* in the liver, suggesting the involvement of these clock components in the impaired daily variability of the lipid metabolic response to LPS. Furthermore, in unstimulated rats, ALAN altered the hepatic expression of *Bmal1*, *Per2*, *Rev-erbα*, *Dbp* and *Nfil3* in a time-of-day-dependent manner compared with the LD regime, indicating a disruption of their daily oscillations.

Adipose tissue plays a central role in linking metabolic and immune responses during inflammation [24]. For instance, endotoxemia is known to stimulate lipolysis and impair insulin sensitivity in adipocytes [4]. Interestingly, we observed a daytime-specific metabolic response to LPS in several parameters in vWAT. LPS-induced *Hsl* expression was higher after daytime than nighttime stimulation, suggesting time-of-day-dependent effects of LPS on adipose lipolysis. A similar response to LPS was observed for *Sirt1* expression, while SIRT1 can further promote fat mobilization in adipocytes [41]. Conversely, daytime LPS reduced adipose *Cpt1b* expression, indicating a decrease in FA oxidation. Similarly, decreased *Glut4* expression was observed only after daytime LPS stimulation, reflecting reduced insulin-dependent glucose uptake and increased insulin resistance in adipose tissue, particularly during immune challenge in the resting period.

In response to LPS, we observed an acute decrease in leptin and adiponectin mRNA levels in vWAT, which paralleled the decrease in their plasma levels. Similarly, an LPS-induced decrease in visceral adiponectin expression was observed in another study in male rats [42]. Conversely, most published studies report stimulatory effects of acute inflammation on leptin in rodents [43, 44], although one study in pigs found reduced adipose leptin expression 6 h after LPS treatment [45], attributing this discrepancy to differences in the use of fasted or fed controls. Thus, the acute decrease in leptin following LPS in our study may be associated with LPS-induced anorexia, as fasting is known to markedly inhibit leptin expression. We found no differences between daytime and nighttime stimulation in the LD regime, but the LPS-induced leptin decrease was specifically eliminated during the day in ALAN rats. Interestingly, this is in line with the attenuated anorectic response to daytime LPS under the ALAN regime, as shown in our previous data [9]. The anorectic response is considered a beneficial sickness symptom and its disruption can adversely impact the immune outcome, especially during bacterial inflammation [46]. In addition, we found that the adiponectin response to LPS was suppressed by ALAN, particularly in the circulation, and this suppression was observed not only after daytime but also after nighttime stimulation.

On the other hand, ALAN led to an upregulation of adipose *Acly* expression after nighttime LPS stimulation, whereas no such response was observed in the LD regime. ACLY catalyses the production of acetyl-CoA, supplying a substrate for lipid biosynthesis and acetylation reactions [47]. In response to LPS, ACLY has been shown to increase in activated macrophages, where it regulates metabolism and the production of inflammatory mediators [48]. Although our data do not allow specific localization of the *Acly* response to adipocytes or adipose macrophages, the results suggest that ALAN exaggerates acetyl-CoA production during nighttime inflammation, thereby fuelling the inflammatory response in vWAT.

The endotoxin-induced metabolic changes in adipose tissue can be mediated by several inflammatory pathways. Our results showed a greater *RelA* and *Nlrp3* response to daytime than nighttime LPS stimulation, pointing out the involvement of the NF-κB/NLRP3 inflammasome pathway. Recent research has been shown that activation of the NLRP3 inflammasome in adipocytes reduces insulin-dependent glucose uptake and contributes to TNF-α-induced insulin resistance [49]. In our previous study, we reported a greater increase in TNF-α levels in rat circulation following daytime than nighttime LPS injection [9], indicating that LPS induction of this proinflammatory cytokine is gated by the circadian clock. Additionally, TNF-α is an important lipolytic factor in adipose tissue [50]. In macrophages, TNF-α has been shown to modulate NAD^+^ metabolism, leading to reduced cellular levels of NAD^+^, a crucial cofactor in redox reactions and for NAD-dependent enzymes [51]. Circadian clocks control NAD^+^ levels through daily oscillations in NAMPT, a rate-limiting enzyme in NAD^+^ biosynthesis [52]. Here, we found that the adipose NAMPT sensitivity to LPS varies by the timing of immune stimulation, with increased *Nampt* expression observed only after daytime LPS. Importantly, disruption of the LD cycle by dim ALAN attenuated the daytime LPS-induced response of the adipose NF-κB/NLRP3 inflammasome pathway and *Nampt* expression, while also suppressing the daytime-specific catabolic response to LPS in vWAT. Together, our data demonstrate that adipose metabolic and inflammatory changes are more responsive to daytime than nighttime endotoxin stimulation and that these day–night differences are compromised by ALAN, supporting the role of circadian regulation. Similar to the liver, ALAN impaired daily variability in the expression of the clock components *Bmal1*, *Per2*, *Rev-erbα* and *Dbp* in vWAT.

Some limitations of our study can be mentioned. We used a sublethal dose of LPS and monitored acute inflammatory and metabolic changes mostly at 3 h post-injection. However, different doses of LPS and multiple sampling times post-injection may provide more complex insights considering the dynamic changes of these LPS-induced responses. Second, as our results were obtained only in male rats, we cannot exclude the possibility of sex-specific responses, which underscores the need for similar studies in females.

In conclusion, our results demonstrate that the time of day of immune stimulation determines distinct adaptations in glucose and FA metabolic pathways specific to the liver and adipose tissue. These metabolic changes were associated with daily variability in the inflammatory response, involving the transcription factor NFIL3 in the liver and the NF-κB/NLRP3 inflammasome pathway in vWAT. The implicated circadian mechanisms were linked to the liver and adipose molecular clocks, which were responsive to endotoxin challenge in a time-of-day-dependent manner. Furthermore, disruption of the LD cycle by dim ALAN attenuated or impaired the daily variability of immune and metabolic responses to LPS, particularly diminishing the capacity to maintain FA metabolic adaptations during inflammation. Taken together, our findings show that the metabolic response triggered by acute inflammation varies with the time of immune activation, which may have important implications for how the body overcomes infection and for optimising therapeutic strategies. Importantly, chronodisruption may deregulate these metabolic and immune interactions during inflammation, potentially leading to immune dysfunction and disease development.

## Electronic supplementary material

Below is the link to the electronic supplementary material.

Supplementary Material 1

## Data Availability

No datasets were generated or analysed during the current study.
