# Supplementary material for: Lipid metabolic adaptations during inflammation are controlled by the circadian clock and impaired by light at night
Source: Inflamm Res. 2025 Jun 30;74(1):96. doi: 10.1007/s00011-025-02066-9 (PMC12206677; doi:10.1007/s00011-025-02066-9)
Supplement: Supplementary file 1 — Supplementary Material 1 [file 11_2025_2066_MOESM1_ESM.pdf]

Supplementary Information

Lipid metabolic adaptations during inflammation are controlled by the circadian clock and impaired by light at night

Beata Benedikova, Viera Sebenova Jerigova, Michal Zeman and Monika Okuliarova*

Department of Animal Physiology and Ethology, Faculty of Natural Sciences, Comenius University, Ilkovicova 6, 842 15 Bratislava, Slovakia

*Corresponding author
E-mail: monika.okuliarova@uniba.sk (MO)

Supplementary Table S1 Primer sequences for real-time PCR

Gene	Accession Number	Forward primer	Reverse primer
Acat1	NM_017075.2	5'-GTCTACCCATTGCCACTCCG-3'	5'-TGACATGCTCTCCATTCCGC-3'
Acly	NM_016987.2	5'-ATGGGCTTCATCGGGCACT-3'	5'-GGCTGCTGGCTCGGTTACAT-3'
Actb	NM_031144.3	5'-GATCAAGATCATTGCTCCTCCTG-3'	5'-AGGGTGTAAAACGCAGCTCA-3'
Adn	NM_144744.3	5'-GCCGTTCTCTTCACCTACGA-3'	5'-CCCCTTCCCCATACACTTGG-3'
Bmal1	NM_024362.2	5'-CACCTTGCGGAATGTCACAG-3'	5'-TACTTCCTTGGTCCACGGGT-3'
Cd36	NM_031561	5'-CGGCGATGAGAAAGCAGAAA-3'	5'-GGCTCATCCACTACTTATTTTCC-3'
Cd68	NM_001031638.1	5'-CTGGGGCCTCTCTGTATTGA-3'	5'-TGATGTCGGTCCTGTTTGAAT-3'
Cpt1b	NM_013200.2	5'-TAAGTGACTGGTGGGAAGAGT-3'	5'-TGCTTGTTGGCTCGTGTTC-3'
Dbp	NM_012543.3	5'-TTGCCCTGTCAAGCATTCCA-3'	5'-ACTTCTCATCCTTCTGTTCCTCG-3'
Fasn	NM_017332.1	5'-GAGTCTGTCTCCCGCTTGAC-3'	5'-TTGCCTTGCTCACCTTCGAG-3'
Foxo1	NM_001191846.2	5'-CAGCAAATCAAGTTATGGAGGA-3'	5'-TATCATTGTGGGGAGGAGAGTC-3'
Glut1	NM_138827.1	5'-GCCGCTTCATCATTGGAGTG-3'	5'-CGAACACCTGGGCAATAAGGA-3'
Glut2	NM_012879.2	5'-GAAGGATCAAAGCCATGTTGG-3'	5'-CCTGATACGCTTCTTCCAGCA-3'
Glut4	NM_012751.1	5'-TATGTTGCGGATGCTATGGGT-3'	5'-AATGTCCGGCCTCTGGTTTC-3'
Hif-1α	NM_024359.1	5'-CAGTTGCCACTTCCCCACAA-3'	5'-TCAATGTCAAGATCACCAGCACC-3'
Hsl	NM_012859.1	5'-TCACGCTACATAAAGGCTGCT-3'	5'-CCACCCGTAAAGAGGGAACT-3'
IL-1β	NM_031512.2	5'-GCCAACAAGTGGTATTCTCCA-3'	5'-GCCGTCTTTCATCACACAGG-3'
Insr	NM_017071.2	5'-CAGTGCCAGTGATGTCTTTCCA-3'	5'-ATTGACCGTCTTCACCGCAA-3'
Lep	NM_013076.3	5'-GACCCCAGCGAGGAAAATGT-3'	5'-GGATACCGACTGCGTGTGT-3'
Lpl	NM_012598.2	5'-TCATCAACTGGTTGGAGGAAG-3'	5'-ACGAAATCCGCATCATCAGG-3'
Mcad	NM_016986.2	5'-GGAGCCGGGACTAGGGTTTA-3'	5'-CTGGCAAACTTCCGAGCAAT-3'
Nampt	NM_177928.3	5'-AAGTTGCTGCCACCTTACCTT-3'	5'-TTCATTCCCTCGACAATCTCCT-3'
Nfil3	NM_053727.2	5'-TTTGACCTGGAGAAGCATGG-3'	5'-GCCTCGGAAACCTTATAGCC-3'
Nlrp3	NM_001191642.1	5'-TCCAGTGTGTTTTCCCAGACC-3'	5'-ACTTGAGAAGAGACCTCGGC-3'
Nr1d1	NM_145775.2	5'-TCCCACATACTTCCCACCATCA-3'	5'-CACTCGGCTGCTGTCTTCCAT-3'
Pck1	NM_198780.3	5'-TTCGGAAGCGGATACGGTG-3'	5'-GCCAGGTTGGTTTTCCCACA-3'
Per2	NM_031678.1	5'-TGGCAACCTTGAAGTACGCT-3'	5'-GCTGGCTCTCACTGGACATT-3'
Pparα	NM_013196.1	5'-GACTAGCAACAATCCGCCTT-3'	5'-GAAGAATCGGACCTCTGCCT-3'
Pparγ	NM_013124.3	5'-TCCAAGAATACCAAAGTGCGA-3'	5'-CCATGAGGGAGTTTGAAGGC-3'
Pygl	NM_022268.1	5'-ATCCACTCGGACATCGTGAA-3'	5'-CCTGGGTTGCAGAGTAAGAG-3'
RelA	NM_199267.2	5'-ATTGTGTTCCGAACTCCCCC-3'	5'-TTTCTTCAATCCGGTGGCGA-3'
Rps29	NM_012876.1	5'-GCTGAACATGTGCCGACAGT-3'	5'-GGTCGCTTAGTCCAACTTAATGAA-3'
Sirt1	NM_012818.2	5'-TTATGCTCGCCTTGCTGTGG-3'	5'-CTGTCCGGGATATATTTCCTTTGC-3'
Srebp-1c	NM_001276707.1	5'-GCCATCGACTACATCCGCTT-3'	5'-CCAGGTCTTTCAGTGATTTGCT-3'
TNF-α	NM_012675.3	5'-TCCGAGATGTGGAACTGGCA-3'	5'-CATTTGGGAACTTCTCCTCCTTGT-3'

Abbreviations: *Acat1*, acetyl-CoA acetyltransferase 1; *Acly*, ATP citrate lyase; *Actb*, actin, beta; *Adn*, adiponectin; *Bmal1*, brain and muscle Arnt-like protein-1; *Cd36*, fatty acid translocase; *Cd68*, lysosome-associated macrophage receptor; *Cpt1b*, carnitine palmitoyltransferase 1b; *Dbp*, D-box binding PAR bZIP transcription factor; *Fasn*, fatty acid synthase; *Foxo1*, forkhead box O1; *Glut*, glucose transporter; *Hif-1α*, hypoxia inducible factor 1 subunit alpha; *Hsl*, hormone sensitive lipase; *Il-1β*, interleukin 1 beta; *Insr*, insulin receptor; *Lep*, leptin; *Lpl*, lipoprotein lipase; *Mcad*, medium chain acyl-CoA dehydrogenase; *Nampt*, nicotinamide phosphoribosyltransferase; *Nfil3*, nuclear factor, interleukin 3 regulated; *Nlrp3*, NLR family, pyrin domain containing 3; *Nr1d1*, nuclear receptor subfamily 1, group D, member 1 (Reverbα); *Pck1*, phosphoenolpyruvate carboxykinase 1; *Per2*, period 2; *Pparα*, peroxisome proliferator activated receptor alpha; *Pparγ*, PPAR gamma; *Pygl*, glycogen phosphorylase L; *RelA*, NF-kB subunit (p65); *Rps29*, ribosomal protein S29; *Sirt1*, sirtuin 1; *Srebp-1c*, sterol regulatory element binding protein 1c; *TNF-α*, tumor necrosis factor alpha

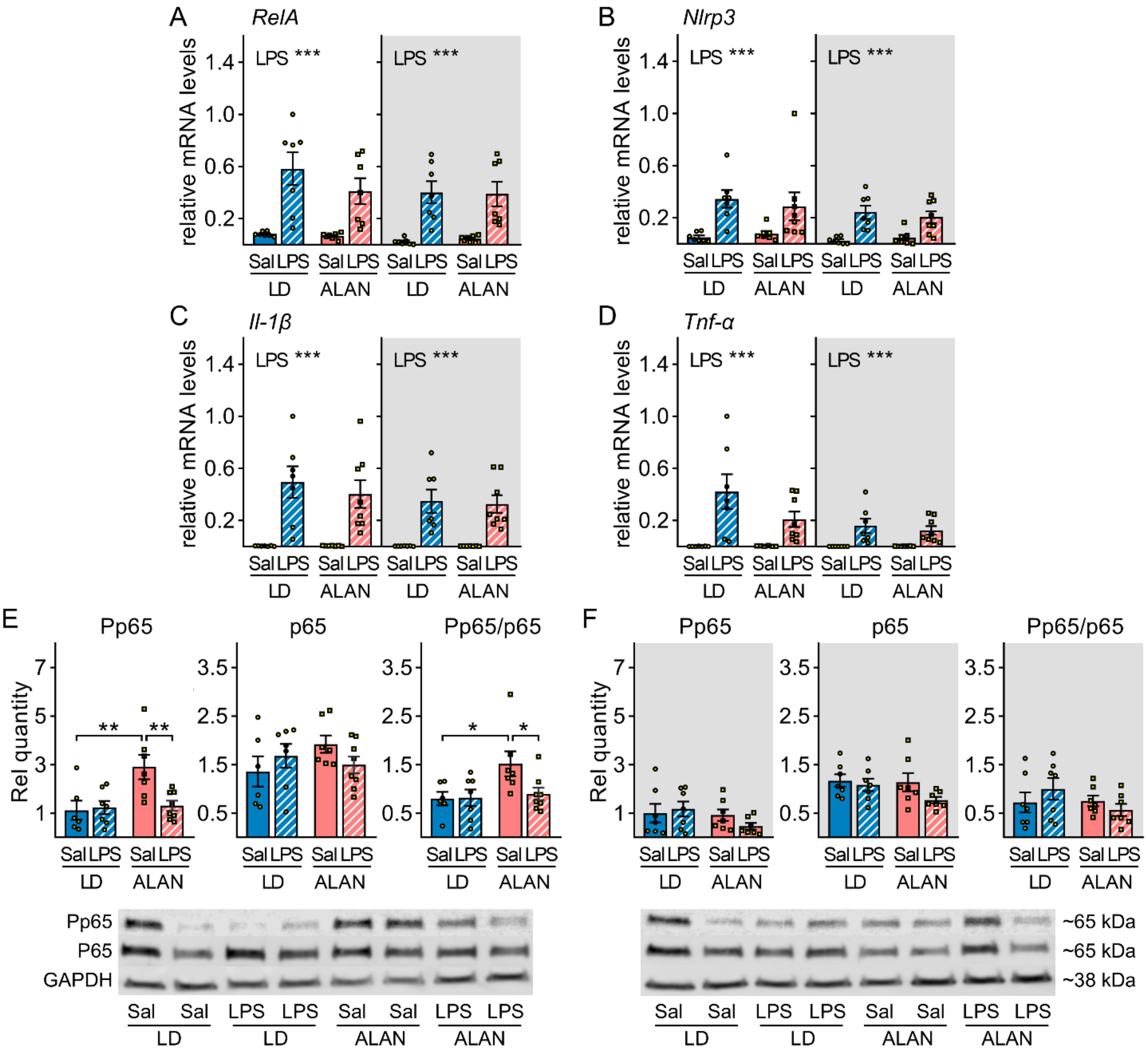

Supplementary Fig. S1 Hepatic inflammatory response to daytime/nighttime lipopolysaccharide (LPS) injection under ALAN. Rats were exposed to either the control 12/12 h light/dark regime (LD) or dim ALAN (~2 lx) and injected with saline (Sal) or LPS at either ZT2 (white background) or ZT14 (shaded background). Zeitgeber time (ZT) 0 = lights on. Data were analysed 3 h post-injection. **A–D** Relative mRNA levels (normalized to *Rps29* and *Actb*) of hepatic *RelA*, NLR family pyrin domain containing 3 (*Nlrp3*), interleukin *Il-1β* and tumour necrosis factor-α (*Tnf-α*). **E–F:** Representative immunoblots and quantification of phosphorylated p65 (Pp65), total p65 and the Pp65/p65 ratio 3 h after daytime (**E**) or nighttime injection (**F**). Bars represent means ± SE (n = 6–8 rats per group). Data were evaluated by two-way ANOVA with Bonferroni's multiple comparisons test at **P* < 0.05, ***P* < 0.01 and ****P* < 0.001

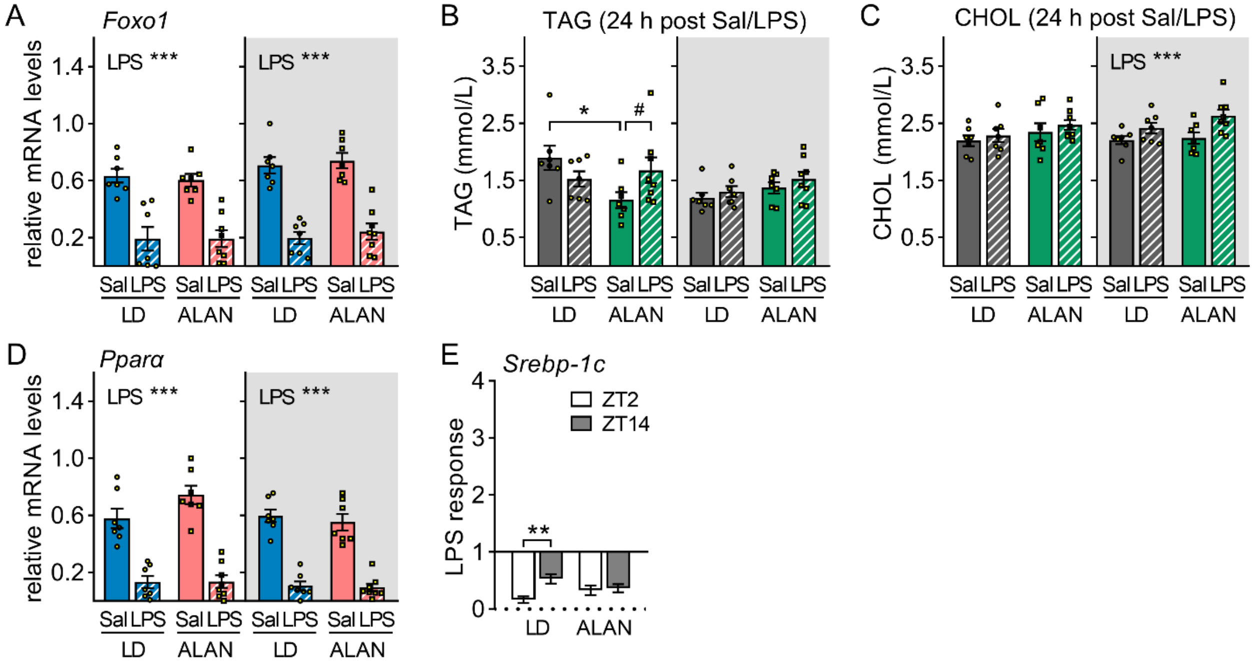

Supplementary Fig. S2 Hepatic metabolic response to daytime/nighttime lipopolysaccharide (LPS) injection under ALAN. Rats were exposed to either the control 12/12 h light/dark regime (LD) or dim ALAN (~2 lx) and injected with saline (Sal) or LPS at either ZT2 (white background) or ZT14 (shaded background). Zeitgeber time (ZT) 0 = lights on. **A** Relative mRNA levels of transcription factor *Foxo1* in the liver 3h post-injection. **B, C** Triacylglycerol (TAG) and total cholesterol (CHOL) plasma levels 24 h post-injection. **D** Relative mRNA levels of peroxisome proliferator-activated receptor-α (*Pparα*) in the liver 3h post-injection. **E** The LPS response (fold change relative to the mean of the saline-injected group) for sterol regulatory element binding protein-1c (*Srebp-1c*). Bars represent means ± SE (n = 6–8 rats per group). Data were evaluated by two-way ANOVA with Bonferroni's multiple comparisons test at $*P < 0.05$, $**P < 0.01$ and $***P < 0.001$

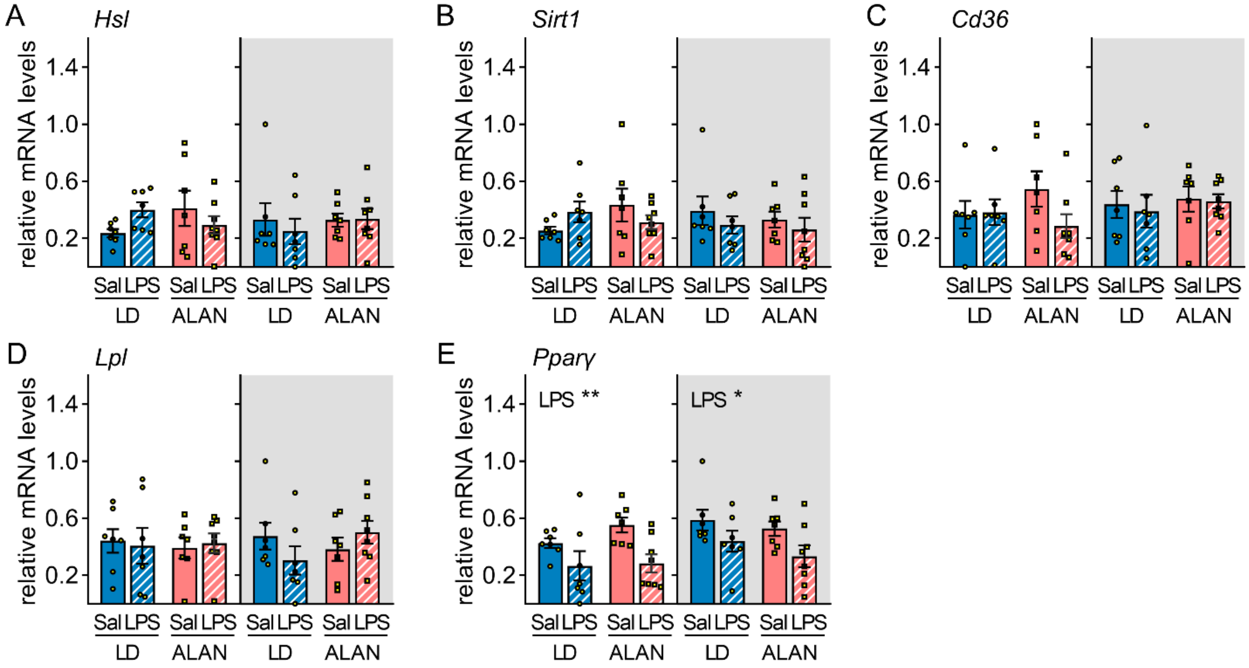

Supplementary Fig. S3 Adipose metabolic response to daytime/nighttime lipopolysaccharide (LPS) injection under ALAN. Rats were exposed to either the control 12/12 h light/dark regime (LD) or dim ALAN (~2 lx) and injected with saline (Sal) or LPS at either ZT2 (white background) or ZT14 (shaded background). Zeitgeber time (ZT) 0 = lights on. Data were analysed in visceral fat (vWAT) 3 h post-injection. Relative mRNA levels of hormone-sensitive lipase (*Hsl*) (**A**), sirtuin 1 (*Sirt1*) (**B**), *Cd36* (**C**), lipoprotein lipase (*Lpl*) (**D**) and peroxisome proliferator-activated receptor-γ (*Pparγ*) (**E**). Bars represent means ± SE (n = 6–8 rats per group). Data were evaluated by two-way ANOVA with Bonferroni's multiple comparisons test at $^{*}P < 0.05$ and $^{**}P < 0.01$

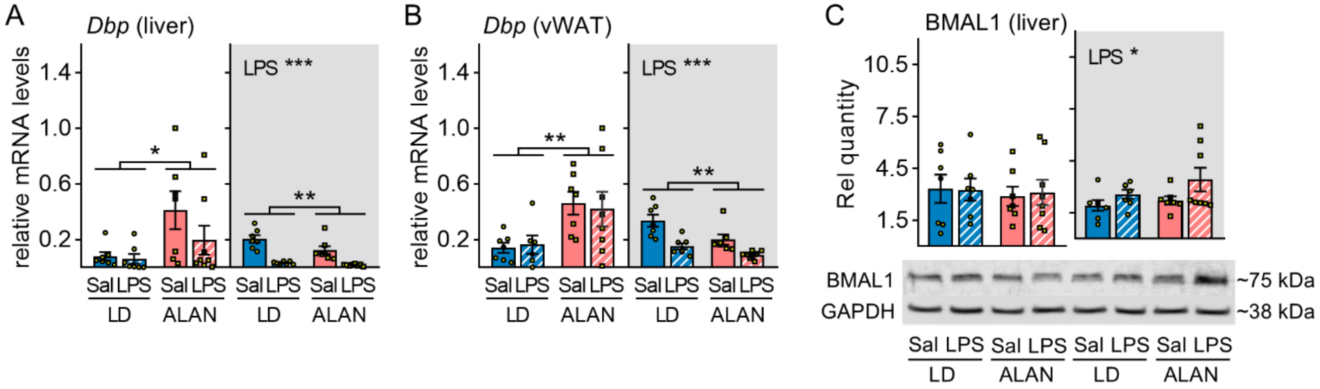

Supplementary Fig. S4 Time-of-day-dependent response of peripheral clock components to LPS-induced inflammation. Rats were exposed to either the control 12/12 h light/dark regime (LD) or dim ALAN (~2 lx) and injected with saline (Sal) or lipopolysaccharide (LPS) at either ZT2 (white background) or ZT14 (shaded background). Zeitgeber time (ZT) 0 = lights on. Relative mRNA levels of D-box binding transcription factor (*Dbp*) in the liver (**A**) and visceral fat (vWAT) (**B**) 3 h post-injection. **C** Protein levels of BMAL1 in the liver and representative immunoblot. Bars represent means ± SE (n = 6–8 rats per group). Data were evaluated by two-way ANOVA with Bonferroni's multiple comparison test at **P* < 0.05, ***P* < 0.01 and ****P* < 0.001
